# Human-Aware Costmap Generation Using Camera–LiDAR Fusion for Mobile Robot Navigation

**DOI:** 10.3390/s26217046

**Published:** 2026-11-07

**Authors:** Wonjin Choi, Jae Hwan Bong, Seongkyun Jeong

**Affiliations:** Department of Human Intelligence and Robot Engineering, Sangmyung University, Cheonan 31066, Republic of Korea; choi11221122@gmail.com

**Keywords:** human-aware navigation, camera–LiDAR fusion, costmap, mobile robot, human detection and tracking

## Abstract

When a mobile robot shares space with people, it should keep extra distance from them rather than treat them as ordinary obstacles. This study proposes a human-aware navigation method that detects a person by fusing a low-cost monocular RGB camera with 2D LiDAR and represents the person in the existing ROS2 Nav2 Obstacle Layer as a body-sized set of virtual obstacle points, without adding a costmap layer plugin. The person’s own LiDAR returns are removed from the scan, so the person is handled only through this body model, while the standard Inflation Layer is left unchanged. In experiments with a real robot and stationary targets, using tape-measured positions as ground truth, the proposed method increased the minimum distance to a person by 48.1% in an open environment and by 22.8% in a narrow environment compared with conventional Nav2, whereas the distance to an ordinary object did not change significantly. Enlarging the inflation radius uniformly for all obstacles gave a similar person distance in the open environment, but it also increased the distance to objects, lowered the success rate, and lengthened the path, and it did not increase the person distance in the narrow environment. When two people stood 0.90 m apart in a 2.4 m passage, the robot could not pass; lowering the inflation radius allowed passage only with negative clearance, which shows a trade-off between clearance and passability. The uniform-inflation comparison used a single setting and does not show an advantage over other human-specific costmap methods, which are compared with only in a literature review. The evaluation was limited to stationary people in a laboratory, a single platform, and a single indoor passage.

## 1. Introduction

Autonomous mobile robots are used in various applications, including logistics, guidance, service, healthcare, and daily assistance, and they increasingly work alongside people as indoor service robots [1]. To operate reliably across these environments, a service robot needs navigation that can accurately perceive its surroundings and move autonomously to a destination [2]. When a robot shares space with people, however, avoiding collisions is not enough; the robot must also account for people’s presence and behavior to move safely and naturally, which is the goal of human-aware navigation [1,3]. Recent studies have explored reactive, proactive, and learning-based methods for socially aware navigation to improve safety and comfort for people, with growing effort to bring predictions of human behavior and social interaction into path planning [3].

Conventional autonomous navigation systems detect obstacles with sensors such as LiDAR and build a costmap to plan a safe path. The widely used ROS2 Navigation2 (Nav2) framework relies on a layered costmap structure, combining an Obstacle Layer and an Inflation Layer to integrate obstacle information and add a safety cost around each obstacle so the planned path avoids collisions [4,5,6]. The Obstacle Layer marks objects detected by LiDAR directly as obstacles in the costmap, while the Inflation Layer extends the cost outward from each obstacle in the same way to keep a safe margin. Building on this layered structure, some studies add new costmap layers to capture specific obstacle properties, representing movable obstacles in a separate layer and adjusting their cost dynamically [6]. A person, however, is not an ordinary obstacle: navigating near people also requires accounting for personal space and social interaction, so a human cost that distinguishes people from other obstacles is needed [1,3,7].

To address this need, human-aware navigation has become an active research area. These studies aim to plan paths that let a robot move around people more safely and naturally by accounting for pedestrians’ personal space and an associated social cost. Proposed approaches include costmap-based methods that assign a social cost around each person, path planning with multi-layer costmaps, and learning-based methods for generating a social cost function; each distinguishes people from ordinary obstacles and produces more human-friendly navigation [8,9,10].

Sensor-based methods for detecting people and estimating their position have also advanced considerably. 2D LiDAR-based tracking offers high ranging accuracy and runs in real time, making it suitable for safe mobile robot navigation, but it cannot easily capture semantic information about a person [11]. RGB-D cameras, in contrast, combine image and depth data to detect and localize people, but they are limited in range and field of view, and their performance can degrade depending on sensor characteristics [12]. Each single-sensor approach has its own strength, either in range accuracy or semantic information, but neither is reliable enough on its own for detecting and localizing people across varied indoor environments.

To overcome this limitation, camera–LiDAR fusion has become a common approach for detecting people [13,14,15,16]. A camera provides appearance and semantic information, while LiDAR provides accurate range, so combining the two improves both detection and position estimation. Building on this complementary information, prior work using camera–LiDAR fusion has improved the robustness of environment perception and object detection beyond what single-sensor methods achieve [13,14,15,16]. Other studies link human detection with navigation to bring social characteristics into path planning, either through a separate navigation module or by generating an additional costmap that represents social distance and human behavior [7,9]. Such approaches add a separate module or costmap to represent people. Whether a person can be distinguished from ordinary obstacles using only Nav2’s standard Obstacle Layer and Inflation Layer, without a dedicated layer or plugin, has received less attention, and this motivates the method studied here.

This study proposes a human-aware navigation method that detects a person and estimates their position by fusing a monocular RGB camera with 2D LiDAR, and represents the person in Nav2’s standard Obstacle Layer as a set of virtual obstacle points on a body-sized circle. The person’s own LiDAR returns are removed from the scan, so the standard Inflation Layer, whose parameters are left unchanged, acts on this body-sized footprint instead of on the small returns from the person’s legs. As a result, a person keeps a larger clearance than an ordinary obstacle without a dedicated costmap plugin. The method is aimed at indoor mobile robots that work near people and must keep a social distance, for example delivery, guide, and serving robots. The validation in this study, however, is limited to stationary people in a laboratory, and use in such settings has not been demonstrated. The method is evaluated on a real mobile robot with stationary targets and compared with conventional Nav2 navigation and with conventional navigation using a uniformly enlarged inflation radius. Performance is assessed with minimum distance, clearance to tape-measured target positions, success rate, path length, and navigation time. The main contribution is a lightweight way to make a standard Nav2 configuration treat people differently from ordinary obstacles. A second contribution is an experimental analysis against tape-measured ground truth that quantifies the added clearance, its cost in path length and time, the comparison with uniform inflation, and the trade-off between clearance and passability in confined spaces. A structured comparison with related studies is given in Section 4.2. No other human-aware method was implemented or tested, and moving people were not evaluated.

## 2. Materials and Methods

Conventional Nav2-based navigation uses a static map and 2D LiDAR to build a costmap of the surrounding environment and plans a path from it. The Global Planner uses the Global Costmap to generate a path from the robot’s current position to the goal, and the Local Planner uses the Local Costmap to determine a driving path that follows the global path while avoiding nearby obstacles. Because a person is detected by 2D LiDAR the same way as any other obstacle, conventional Nav2 does not distinguish a person from surrounding obstacles when building the costmap.

**Figure 1:**
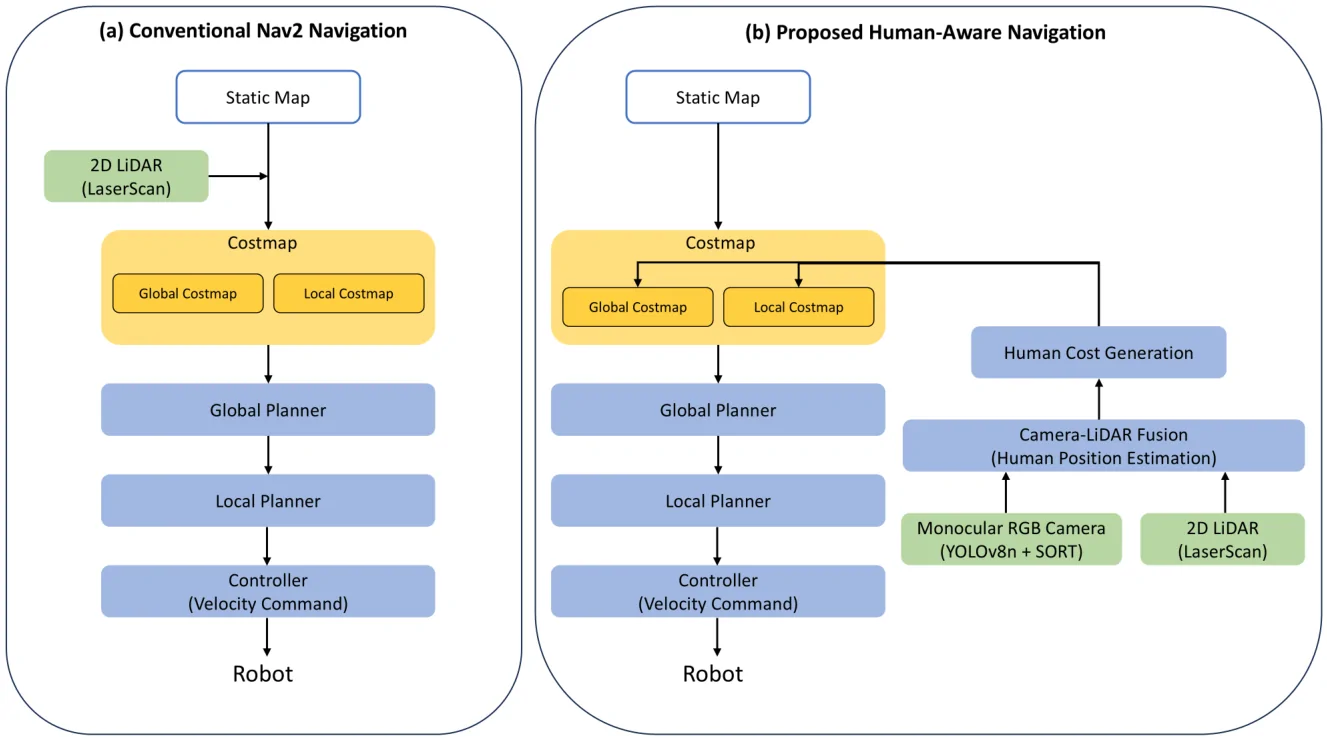
Comparison of the conventional Nav2 architecture and the proposed human-aware navigation approach.

This study proposes a human-aware navigation method that keeps Nav2’s path generation and tracking structure intact and changes how a detected person enters the costmap. A monocular RGB camera and 2D LiDAR first detect a person and estimate their position. The person is then represented as a body-sized obstacle in the Obstacle Layer, and the person’s own LiDAR returns are removed from the scan. The standard Inflation Layer adds cost around this obstacle in the same way as around any other obstacle. The resulting obstacle region and the cost around it are called human cost in this paper. Figure 1 compares the overall process of conventional Nav2-based navigation with that of the proposed method.

### 2.1. Hardware Configuration

**Figure 2:**
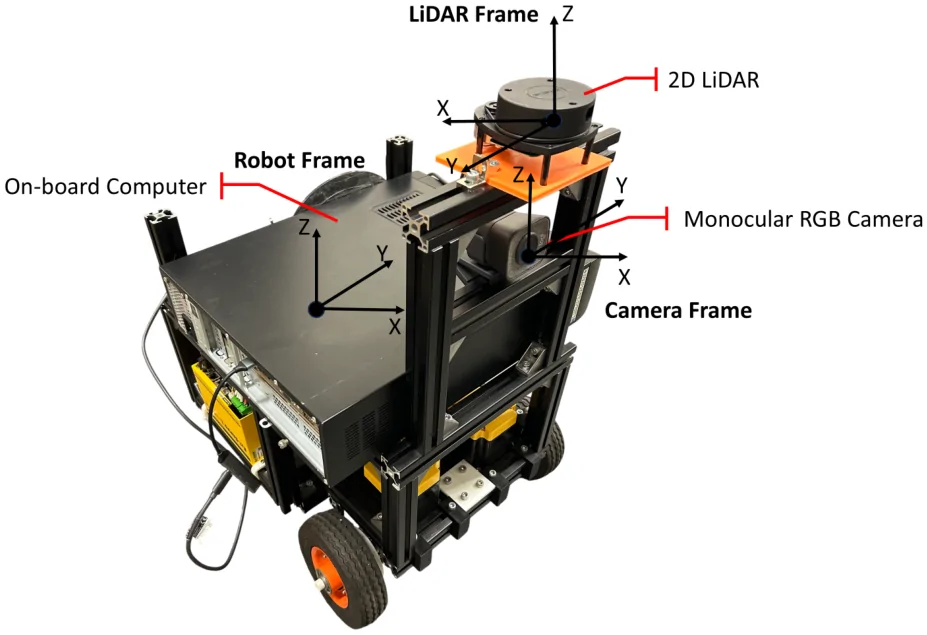
Hardware configuration and sensor coordinate frames of the mobile robot.

Figure 2 shows the hardware configuration and sensor coordinate frames of the mobile robot platform used in this study. The robot uses a differential-drive design and measures 420 mm wide, 480 mm long, and 660 mm high. A 2D LiDAR is mounted at the top front of the robot to measure the distance to surrounding objects, and a monocular RGB camera is mounted below it to capture image data. Relative to the robot’s reference frame, the camera is located at (0.24, 0, 0.36) m and the LiDAR at (0.24, 0, 0.48) m. The two sensors therefore share the same horizontal position and differ only by 0.12 m in height. The LiDAR frame follows the coordinate convention defined by the sensor manufacturer, so its *x*-axis points opposite to that of the robot frame and the camera frame. Both sensors face the robot’s direction of travel, so their fields of view cover the same forward region.

**Figure 3:**
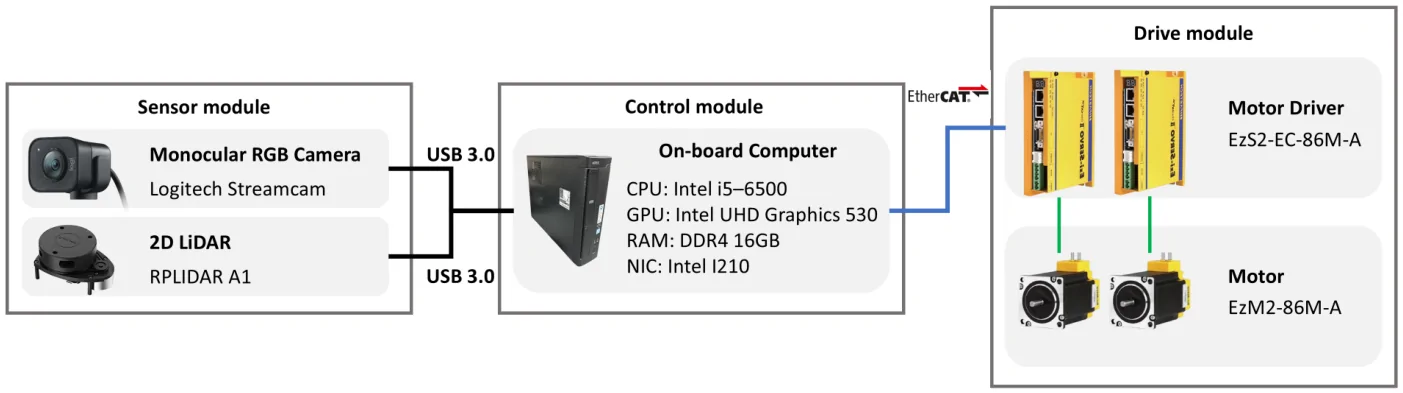
Hardware architecture of the proposed mobile robot.

Figure 3 shows the hardware configuration of the mobile robot and the connections between its components. The sensor module consists of the monocular RGB camera and the 2D LiDAR, both connected to the on-board computer through USB 3.0.

The on-board computer, mounted on top of the robot, processes the data from each sensor and performs the computation needed for sensor fusion, path planning, and robot control. It sends the resulting motion commands to the motor drivers over EtherCAT, and the motor drivers control the drive motors accordingly. Table 1 lists the detailed hardware specifications.

### 2.2. Software Framework of the Proposed System

The proposed human-aware navigation system combines information from the monocular RGB camera and the 2D LiDAR to estimate a person’s position, then feeds the resulting human cost into Nav2’s path planning.

**Figure 4:**
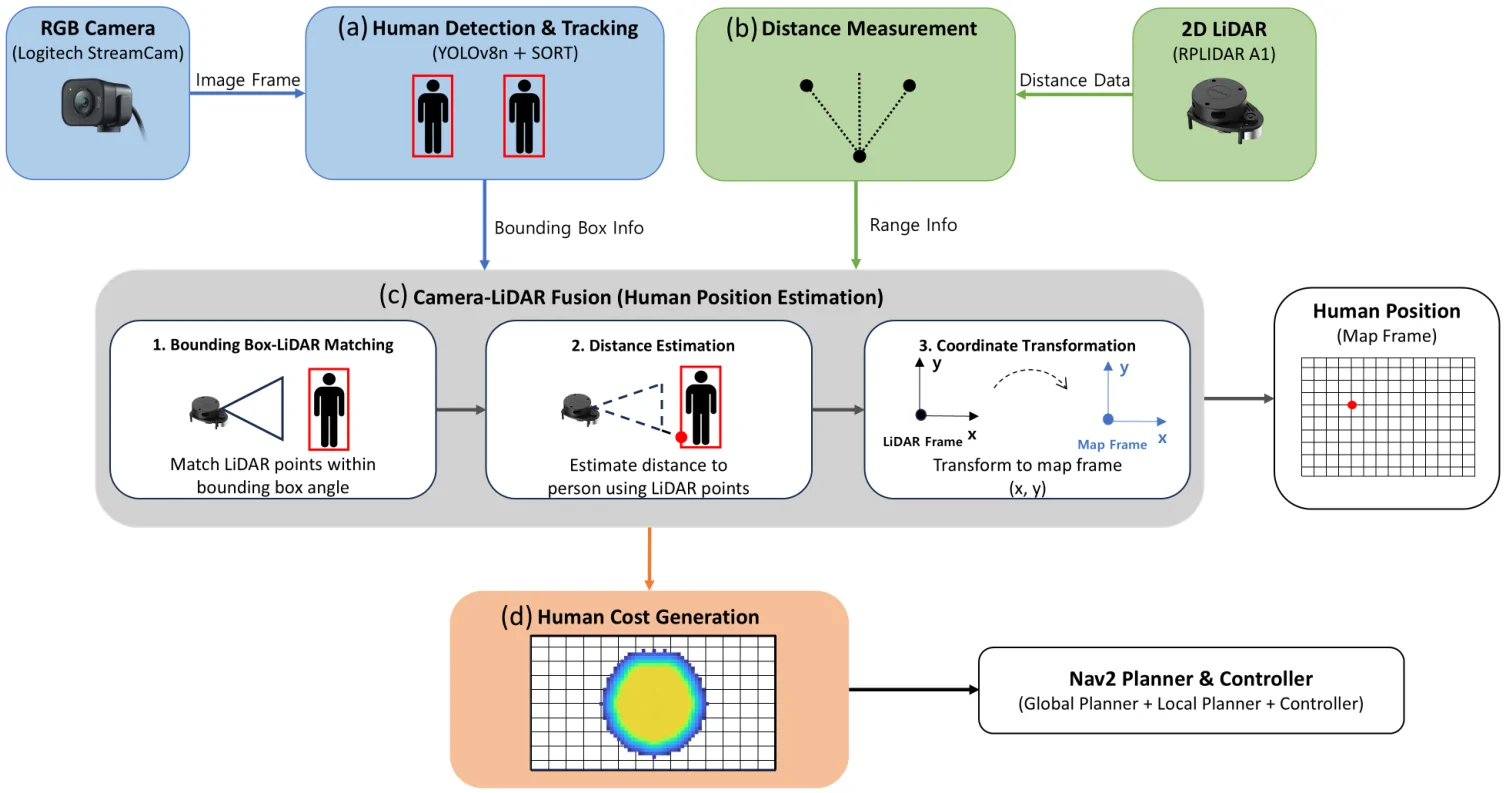
Overall software architecture and data flow of the proposed human-aware navigation system.

Figure 4 shows the overall system architecture and data flow, consisting of human detection and tracking, LiDAR range acquisition, camera–LiDAR fusion for position estimation, human cost generation, and navigation, in that order. This keeps Nav2’s path planning and driving structure intact while adding information about people. Each step is described in the following  sections.

#### 2.2.1. Human Detection Using YOLO and SORT

Figure 4a shows the process of detecting and tracking a person using the monocular RGB camera. YOLOv8n is applied to each image frame to detect a person and obtain their location as a bounding box. SORT (Simple Online and Realtime Tracking) then tracks the same person across consecutive frames. The resulting bounding box is later used as input to camera–LiDAR fusion.

YOLOv8n is the most lightweight model in the YOLOv8 family, offering fast inference even with limited computing resources [17]. Because the mobile robot must process sensor data and plan its path at the same time, keeping up a steady stream of detection results matters as much as accuracy, so this study uses YOLOv8n for human detection. Detection runs on the on-board computer’s CPU alone, without GPU acceleration. With navigation, camera–LiDAR fusion, and human detection and tracking all running together, the update rate for detection and tracking measured about 7.6 Hz (Section 3.6), showing that the system can keep refreshing these results and running navigation even on limited hardware.

Detections from a single frame alone can produce an unstable bounding box when a person moves or is briefly missed. SORT addresses this by tracking the same person across consecutive frames: it predicts each object’s state with a Kalman filter and matches detections to existing tracks using the Hungarian algorithm to keep each object’s ID consistent [18]. This keeps the bounding box stable and supports more reliable position estimation during camera–LiDAR fusion.

#### 2.2.2. 2D LiDAR-Based Distance Measurement

Figure 4b shows how range data is obtained from the 2D LiDAR. The LiDAR scans the surrounding environment and returns a distance for each scan angle, giving the range to an object in any given direction.

This study does not use the 2D LiDAR to detect people; instead, it measures the distance to a person already detected by the RGB camera. The LiDAR range value at the angle corresponding to the detected person is used for this purpose. The specific method for combining camera and LiDAR data to compute this distance is described in the next section on camera–LiDAR fusion.

#### 2.2.3. Camera–LiDAR Fusion-Based Human Position Estimation

Figure 4c shows the process of estimating a person’s position through camera–LiDAR fusion. The bounding box from YOLOv8n and SORT is combined with LiDAR range data to estimate the person’s position. The center pixel of the bounding box is first computed and converted to a horizontal viewing angle in the camera frame, and the LiDAR range at the corresponding angle then gives the distance to the person.

The horizontal viewing angle is computed from the center pixel of the detected bounding box. Given the left and right boundary coordinates of the bounding box, $x_{min}$ and $x_{max}$, the center pixel’s horizontal coordinate $u_{c}$ is given by Equation (1).

(1) $$u_{c}=\frac{x_{min}+x_{max}}{2}$$

The center pixel coordinate $u_{c}$ is then converted to a horizontal viewing angle in the camera frame using the camera’s intrinsic parameters. These parameters and the lens distortion coefficients were obtained through OpenCV camera calibration. Only the horizontal focal length $f_{x}$ and the horizontal principal point $c_{x}$ are used for this calculation, and no separate lens distortion correction is applied to the image. The horizontal viewing angle $\theta_{c}$ corresponding to $u_{c}$ is then given by Equation (2).

(2) $$\theta_{c}=\tan^{-1}\;\left(\frac{u_{c}-c_{x}}{f_{x}}\right)$$

To match the viewing angle $\theta_{c}$ from the camera with LiDAR range data, the orientation relationship between the two sensor frames shown in Figure 2 must be taken into account. Because the two sensors’ angle references differ by 180° (π rad), the LiDAR angle $\theta_{l}$ corresponding to $\theta_{c}$ is given by Equation (3).

(3) $$\theta_{l}=\pi -\theta_{c}$$

To select the range value at $\theta_{l}$, the corresponding index *i* in the LiDAR scan must be identified. Since the LiDAR samples range data at a fixed angular interval, index *i* is computed from the scan’s starting angle $\theta_{min}$ and the angular interval Δθ between consecutive measurements, as given by Equation (4).

(4) $$i=\frac{\theta_{l}-\theta_{min}}{\Delta \theta }$$

Index *i* from Equation (4) is rounded to the nearest integer to select the closest LiDAR measurement direction. Because the two sensors’ angle references differ, the resulting index can fall outside the valid scan range; this is corrected using the fact that a 360° scan wraps around, so the start and end of the scan connect to the same measurement direction. Given the total number of scan points *N*, an index outside the valid range 0≤i<N is mapped back to the corresponding valid index this way.

To reduce the effect of small mismatches between the camera’s viewing direction and the LiDAR’s measurement directions, along with momentary fluctuations in range readings, the range values around the selected direction are also considered together rather than relying on a single measurement. This reduces instability in the distance estimate and yields the range value used for the person’s viewing direction.

To further smooth out momentary jumps in range during continuous measurement, each new range value is also compared with the previous one. A difference greater than 0.6 m is treated as an abrupt change, in which case the nearby LiDAR range values are searched for one within 0.3 m of the previous measurement, and that value is used instead. This limits the effect of momentary range fluctuations on the person’s estimated distance. To check how sensitive this correction is to the 0.6 m threshold, the threshold was varied between 0.4 m and 0.8 m and the resulting position error compared; the difference in error across this range was small, so 0.6 m, the midpoint of the tested range, was adopted as the threshold. The 0.3 m recovery margin is applied only when an abrupt change is detected.

Using the resulting distance *r* to the person and the LiDAR angle $\theta_{l}$, the person’s position $\left(x_{p},y_{p}\right)$ in the robot frame is given by Equations (5) and (6).

(5) $$x_{p}=r\cos\theta_{l}$$

(6) $$y_{p}=r\sin\theta_{l}$$

The relative position $\left(x_{p},y_{p}\right)$ is then converted to the map frame used for navigation, based on the robot’s position and orientation, giving the person’s position $\left(x_{h},y_{h}\right)$ in the map frame. This position is used as input for generating the human cost. Two properties of this estimate matter for the evaluation. First, the LiDAR scan plane is 0.48 m above the floor, so the measured range refers to the surface of the person’s legs facing the robot, and the selected return can change with the person’s posture. Second, only the camera intrinsic parameters were calibrated. No extrinsic calibration between the camera and the LiDAR was performed; the two sensors share the same horizontal mounting position, and their alignment relies on the mechanical mounting, which was checked in the camera image and with tape-measured distances. Any residual angular misalignment is therefore included in the angle errors reported in Section 3.1. An angular error of 1.2° corresponds to a lateral error of about 0.04 m at a range of 2 m.

#### 2.2.4. Human Cost Generation

The person’s position estimated through camera–LiDAR fusion is used to represent the person in Nav2’s Obstacle Layer. In this paper, human cost refers to the obstacle region generated from the person’s body model together with the cost that the standard Inflation Layer assigns around it.

The person’s position $\left(x_{h},y_{h}\right)$ in the map frame is used as the reference. Because representing a person as a single point cannot capture the space their body occupies, the person’s body is modeled as a circle [19,20]. A set of obstacle points is generated along the circumference of this circle, centered on the person’s position; each point’s coordinates $\left(x_{i},y_{i}\right)$ are given by Equations (7) and (8), using the person’s position $\left(x_{h},y_{h}\right)$, radius *R*, and angle $\theta_{i}$.

(7) $$x_{i}=x_{h}+R\cos\theta_{i}$$

(8) $$y_{i}=y_{h}+R\sin\theta_{i}$$

Here, $\left(x_{i},y_{i}\right)$ is the coordinate of the *i*-th obstacle point representing the person’s body, *R* is the radius of the circular body model, and $\theta_{i}$ is that point’s angle. The radius *R* was set to 0.30 m. This is half of the 0.60 m long axis of the 0.50 m × 0.60 m body ellipse that the Federal Highway Administration recommends for pedestrian standing areas [21], which is a simplification of the body ellipse of Fruin [22]. The circle that encloses this ellipse was used as the body model. The value 0.30 m is therefore a modeling radius for generating the human cost, and not a measured body dimension. It is larger than the half-width of a person measured in the LiDAR scan, which was about 0.20 m in our setup. The points are published as a point cloud (/person_points) and registered as a second observation source of the Obstacle Layer, in addition to the LiDAR scan. This source is configured for marking only (marking enabled, clearing disabled), with a maximum obstacle range of 3.0 m and an observation persistence of 0 s; no expected update rate was set. The publication rate of the point cloud is reported in Section 3.6.

The person’s own LiDAR returns are handled separately. For each tracked bounding box, the horizontal extent of the box is converted to the corresponding LiDAR angles with the same mapping as in Equations (2)–(4), and the ranges in that angular sector are set to +∞ in a copy of the scan (/scan_without_people), which the scan observation source uses instead of the raw scan. The Obstacle Layer option that accepts infinite ranges as valid (inf_is_valid) is enabled, so these beams are treated as directions without a detected obstacle. The width of the removed sector follows the bounding box and therefore varies with the person’s apparent size. When no tracked bounding box is published for a frame, the raw LiDAR returns are retained and the person is treated as an ordinary obstacle until detection resumes.

**Figure 5:**
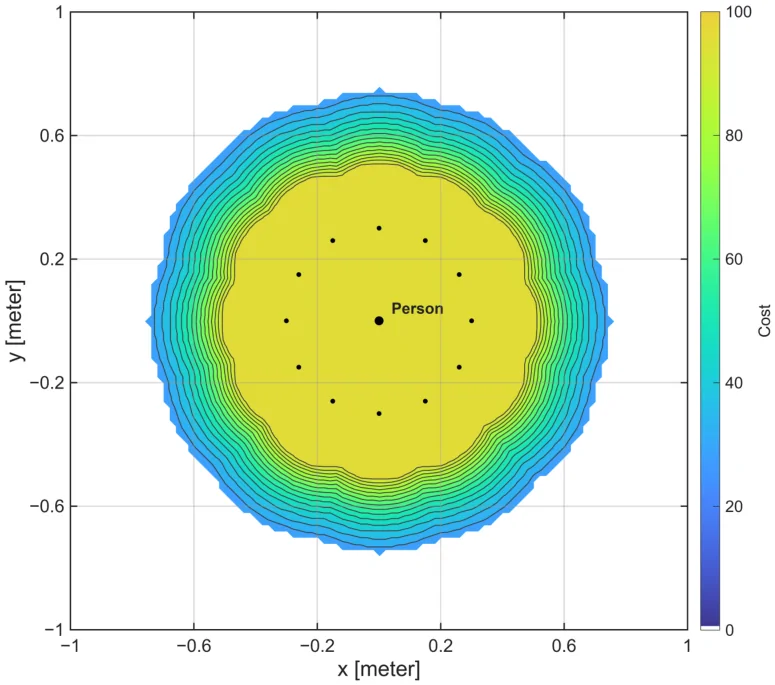
Generated human cost distribution. Black dots indicate the discretized boundary points of the occupied region computed from Equations (7) and (8).

The Inflation Layer is not person-specific. It uses an inflation radius of 0.45 m and a cost scaling factor of 5.0 in both the Global and Local Costmaps, and these values apply to every obstacle, including the virtual person points. Where the inflated costs of different points overlap, the higher value is kept rather than summed. The cost around a person therefore extends about 0.75 m from the person’s center (the 0.30 m body radius plus the 0.45 m inflation radius). The 0.45 m decay itself is a shared setting and not a person-specific parameter. What distinguishes a person from an ordinary obstacle is the footprint to which this inflation is applied, namely a body-sized circle instead of the small returns from the legs that the LiDAR alone would provide. Figure 5 shows the spatial distribution of human cost centered on a person’s position.

### 2.3. Experimental Method

The proposed human-aware navigation system was evaluated in two parts: validating the position estimation accuracy of camera–LiDAR fusion, and validating the navigation performance of human-aware navigation. Camera–LiDAR fusion was first validated by comparing the estimated position of a person with their actual position. Navigation experiments were then conducted across different spatial configurations and target arrangements to evaluate how human cost affects path planning and driving behavior. Each experiment’s configuration and evaluation method are described below.

#### 2.3.1. Validation Experiments for Camera–LiDAR Fusion

To validate the distance and angle estimated through camera–LiDAR fusion, measurements were taken of a stationary person in an open space free of surrounding obstacles. The person was placed at 0° and 20° relative to the robot’s forward direction, with distance measured from the LiDAR’s mounted position to the midpoint between the person’s feet, measured with a tape measure. At each angle, distance was varied from 0.5 m to 2.0 m in steps of 0.5 m, giving eight measurement conditions in total.

Ten repeated measurements were taken at each distance-angle combination, with camera–LiDAR fusion used to obtain the estimated distance and viewing angle each time. This gave 80 measurements in total, across four distance conditions and two angle conditions with ten repetitions each.

Accuracy was evaluated from the difference between each measurement and its ground-truth value. For each of the ten repeated measurements at a given distance and angle, the distance error and angle error relative to the ground truth were computed, and their mean and standard deviation were used to assess measurement accuracy and repeatability.

#### 2.3.2. Single-Target Navigation Scenario

A single-target navigation experiment was conducted to examine how the proposed human cost affects the mobile robot’s path planning and driving around a single target. The conventional Nav2-based method is referred to as Conventional, and the proposed method with human cost applied is referred to as Proposed. All robot and navigation settings were kept identical between the two methods except for the presence of human cost.

The single-target experiment used two environments, an open environment and a narrow environment, differing in how much space the robot had to avoid the target. An object or a person was placed alone in each environment, and both Conventional and Proposed were tested in each case. The target remained stationary throughout every trial, and the object used was similar in size to a human body. Each condition was repeated ten times in the same environment. Table 2 summarizes the single-target navigation experiment.

**Figure 6:**
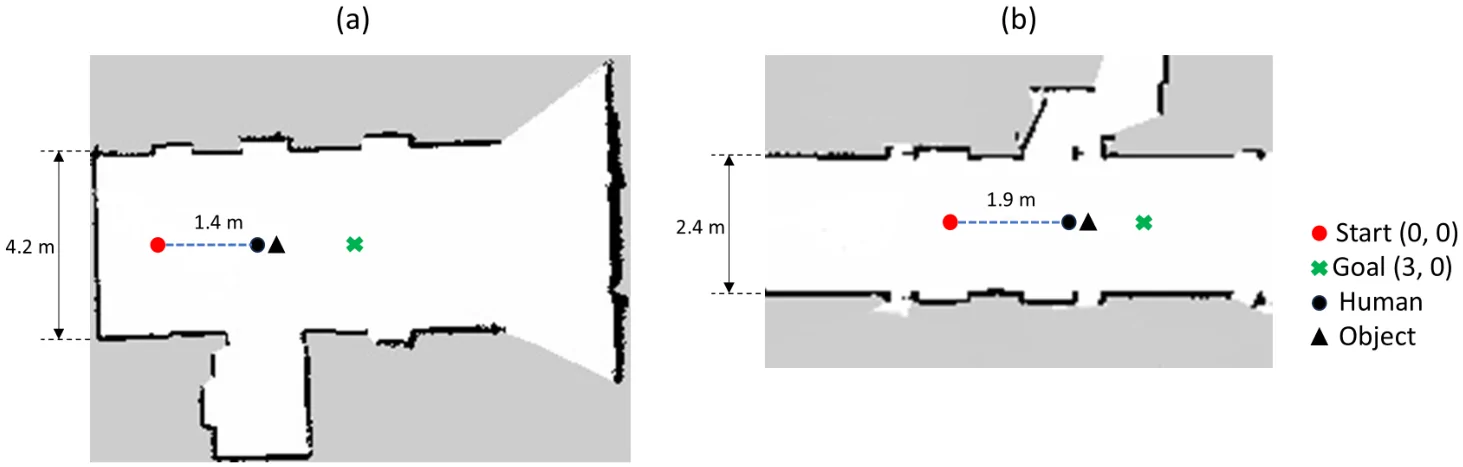
Experimental configurations for single-target navigation: (**a**) Open Environment and (**b**) Narrow Environment.

Figure 6 shows the environment and target placement for the single-target navigation experiments. Figure 6a shows the open environment and Figure 6b the narrow environment. The red circle marks the robot’s starting position, the green X marks the goal, and the black circle and triangle mark the positions of the person and the object, respectively. The robot’s start and goal positions were set to (0, 0) and (3, 0) in the map frame, with its initial orientation facing the goal. Navigation was considered complete once the robot came within 0.5 m of the goal in every experiment.

In the open environment shown in Figure 6a, the driving space was 4.2 m wide to give the robot enough room to avoid the target, which was placed 1.4 m from the robot’s starting position in the direction of the goal. In the narrow environment shown in Figure 6b, the driving space was 2.4 m wide to limit the room available for avoidance, and the target was placed 1.9 m from the starting position in the direction of the goal. The person and object shown together in Figure 6 were not present at the same time; each was placed independently at the position shown for its respective target condition.

The target positions were measured with a tape measure from the map origin. In the open environment the target was placed at (1.40, 0.00) m, and in the narrow environment at (1.90, 0.00) m. For a person, this position is the midpoint between the feet; for the object, it is the center of its footprint (0.30×0.30 m, height 0.68 m). Within each condition, the ten Conventional trials were performed first, and the ten Proposed trials followed in the same session; the order of trials was not randomized. A trial was counted as a success when Nav2 returned SUCCEEDED and as a failure when it returned ABORTED.

#### 2.3.3. Multi-Target Navigation Scenario

In addition to the single-target experiments, a multi-target experiment was designed to examine how human cost is reflected in navigation when multiple targets are close together. This experiment was conducted in the narrow environment, where avoidance space is limited, and covered two conditions: a human-object condition and a human-human condition. Proposed was used for both conditions, and each target remained stationary throughout the experiment.

In the human-object condition, a person and an object were placed close enough that their surrounding cost regions could interact, with a center-to-center distance of 1.25 m. In the human-human condition, two people were placed so that their human cost regions overlapped, with a center-to-center spacing of either 0.45 m or 0.90 m to examine how this spacing affects the driving space available to the robot. All positions were measured with a tape measure. In the human-object condition, the person stood at (2.00,−0.30) m and the object at (2.00, 0.95) m. In the human-human conditions, the two people stood along x=1.95 m, at y=0.45 m and y=0.00 m in the 0.45 m condition and at y=±0.45 m in the 0.90 m condition. The 0.45 m layout is therefore not symmetric about the center line of the passage, and the free width differs on the two sides of the pair.

**Figure 7:**
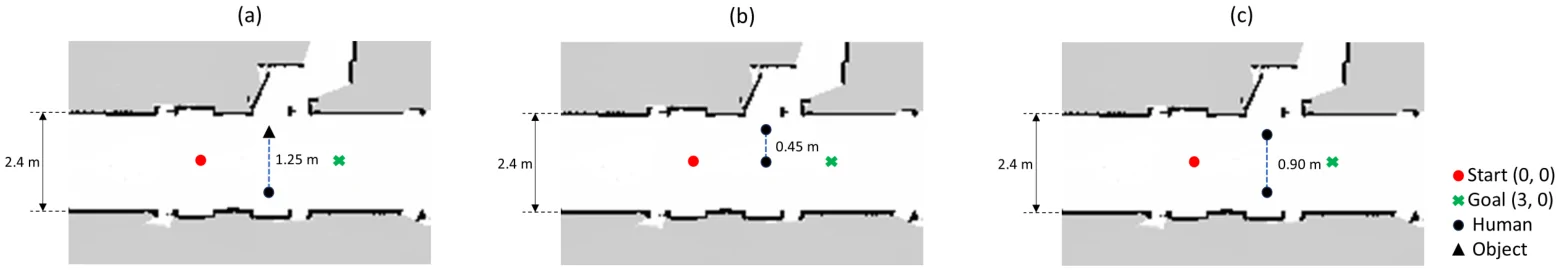
Experimental configurations for multi-target navigation: (**a**) Human-Object, (**b**) Human-Human with a distance of 0.45 m, and (**c**) Human-Human with a distance of 0.90 m.

Figure 7 shows the environment and target placement for the multi-target navigation experiments. Figure 7a shows the human-object condition, and Figure 7b,c show the human-human condition with center-to-center distances of 0.45 m and 0.90 m. The robot’s start and goal positions, initial orientation, and completion criterion were the same as in the single-target experiments, and all experiments were conducted in the narrow environment, 2.4 m wide.

#### 2.3.4. Navigation Performance Evaluation Metrics

Navigation time, path length, and minimum distance to the target were used to quantitatively evaluate driving performance [23], and a clearance measure defined below was added to describe the margin to the target. The driving trajectories recorded across repeated trials were also compared to examine differences in path shape between Conventional and Proposed.

Navigation time *T* was computed as the elapsed time from when navigation started, $t_{s}$, to when the driving outcome was determined, $t_{e}$, as given in Equation (9).

(9) $$T=t_{e}-t_{s}$$

Path length *L* was computed from the robot’s recorded positions in the map frame. Given *N* recorded positions during a trial and the robot’s *i*-th position $\left(x_{r,i},y_{r,i}\right)$, the Euclidean distance between consecutive positions was summed over the entire trial, as given in Equation (10).

(10) $$L=\sum_{i=1}^{N-1}\sqrt{{\left(x_{r,i+1}-x_{r,i}\right)}^{2}+{\left(y_{r,i+1}-y_{r,i}\right)}^{2}}$$

Minimum distance to the target, $D_{min}$, was computed from the robot’s recorded positions and the target’s position during each trial. Given the robot’s *i*-th position $\left(x_{r,i},y_{r,i}\right)$ and the target’s position $\left(x_{t},y_{t}\right)$, the Euclidean distance between them was computed at each recorded position, and the smallest value over the trial was taken as $D_{min}$, as given in Equation (11).

(11) $$D_{min}=\min_{1\leq i\leq N}\sqrt{{\left(x_{r,i}-x_{t}\right)}^{2}+{\left(y_{r,i}-y_{t}\right)}^{2}}$$

For the person condition, the target position was the midpoint between the person’s feet, measured with a tape measure, which is used as ground truth throughout the navigation analysis. For the object condition, the target position was the center of the object as placed in the experiment. The position estimated by camera–LiDAR fusion was not used to evaluate the distance.

Mean and standard deviation of navigation time, path length, and minimum distance were computed over the successful trials of each condition. Trials that ended with an ABORTED result were counted as failures and excluded from these statistics, and the success rate counts all ten trials. Driving trajectories were plotted from the robot’s recorded positions in the map frame, overlaying the trials of each condition. The statistical significance of differences between Conventional and Proposed was assessed with Welch’s independent *t*-test on the successful trials, with a significance level of α=0.05.

To describe the margin between the robot and a target, a clearance was computed from $D_{min}$. The robot footprint is a rectangle with corners at (±0.24,±0.21) m, whose circumscribed radius is $r_{r}=0.319$ m. The target was also approximated by a circle: $r_{t}=0.30$ m for a person, which is the radius of the body model, and $r_{t}=0.212$ m for the object, which is the circumscribed radius of its 0.30×0.30 m footprint. The clearance *C* is given by Equation (12).

(12) $$C=D_{min}-\left(r_{r}+r_{t}\right)$$

A clearance of zero or less is called an overlap of the circumscribed circles, and a clearance between 0 and 0.10 m is called a near-miss. Overlap and near-miss are model-based quantities. They are computed from circles and tape-measured positions, and they are not observed physical events. Observed physical contact was recorded separately (see below). The 0.10 m threshold is a descriptive choice of the same order as the position differences between the estimated and tape-measured positions reported in the following sections  the number of near-misses depends on it. For each condition, the worst case is the smallest $D_{min}$ among the successful trials, and the mean and minimum clearance are computed from the per-trial values of *C*. In multi-target conditions, the clearance was computed separately for each target. Overlap is a conservative geometric indicator and does not mean physical contact. Both shapes are replaced by circles, and the radius of the person model (0.30 m) is larger than the measured half-width of the body (about 0.20 m). Whether the robot touched a target was checked visually by the experimenters during the trials; this check was not recorded on video for the main experiments.

#### 2.3.5. Uniform Inflation Baseline

The proposed method increases the clearance from a person by changing the footprint to which the standard inflation is applied. A simpler alternative is to enlarge the inflation radius of conventional Nav2 for all obstacles. To test whether this alternative has a similar effect, Conventional was repeated with the inflation radius increased from 0.45 m to 0.78 m, with the raw LiDAR scan and no person-specific processing. This condition is called Uniform Inflation. The value of 0.78 m was selected in preliminary runs so that the minimum distance to a person in the open environment was close to that of Proposed. It was not tuned for the other conditions, so the comparison represents a single setting that is matched to Proposed in one condition. The four single-target conditions (open and narrow environments, object and person) were each repeated ten times, with the same target positions and metrics as above. The inflation radius of 0.78 m was applied to both the Global and the Local Costmaps, the cost scaling factor remained 5.0, and the distances were computed from the tape-measured target positions, in the same way as for Conventional and Proposed.

#### 2.3.6. Inflation Radius Variation in the Two-Person Passage

To examine the trade-off between clearance and passability, the condition in which Proposed failed in all trials (two people with 0.90 m spacing) was repeated with a smaller inflation radius of 0.40, 0.35, and 0.30 m. Each setting was run five times with Proposed, keeping the body-model radius (R=0.30 m), the cost scaling factor (5.0), and the other settings unchanged. The number of successful passes between the two people was recorded. A smaller body-model radius *R* was not tested.

#### 2.3.7. Measurement of Computational Load

To characterize the computational load, the CPU and memory use of each software component were measured with pidstat over 120 s of navigation with Proposed, and the publication rates of the main topics were measured with ROS 2 tools. The on-board computer is the one listed in Table 1, with four logical CPUs and no GPU acceleration. The end-to-end latency from image capture to the appearance of the person in the costmap was not measured, because it requires timestamping inside each node and in the costmap, which was not implemented.

#### 2.3.8. Data Provenance and Statistical Treatment

The distances in all navigation results were computed from the tape-measured positions of the people and objects. A trial was counted as a success when the robot reached the goal, and a trial that ended with the Nav2 result ABORTED was counted as a failure. In the navigation results of Conventional, Proposed, and Uniform Inflation, navigation time, path length, minimum distance, clearance, worst case, and the Welch tests were computed from the successful trials only, and the success rate was calculated over all ten trials of each condition. The position estimation results during navigation were computed from all ten trials of each row, because a position estimate is available in every trial. The Conventional and Proposed rows in the comparison with Uniform Inflation are the same as those of the single-target navigation results. The uniform-inflation trials, the inflation sweep, and the computational load measurements were performed as separate experiments.

## 3. Results

### 3.1. Camera–LiDAR Fusion Validation

To evaluate the distance and angle accuracy of camera–LiDAR fusion, measurements were analyzed under the distance and angle conditions defined in Section 2.3.1. Ten repeated measurements were taken at each distance-angle condition, and the mean estimated distance and viewing angle were computed. The absolute error between each measurement and its ground-truth value was then computed, and the mean and standard deviation of distance error and angle error were derived from these values. Table 3 shows the results at 0°.

At 0°, the mean distance error ranged from 0.019 to 0.022 m, and the mean angle error ranged from 1.18° to 1.32°. Both errors remained at a similar level across all four distances.

To check the results off-axis, the same distance conditions were measured with the person placed at 20°. Table 4 shows these results.

At 20°, the mean distance error ranged from 0.020 to 0.023 m, and the mean angle error ranged from 1.16° to 1.31°, close to the values obtained at 0°. Neither error increased or decreased consistently with distance.

**Figure 8:**
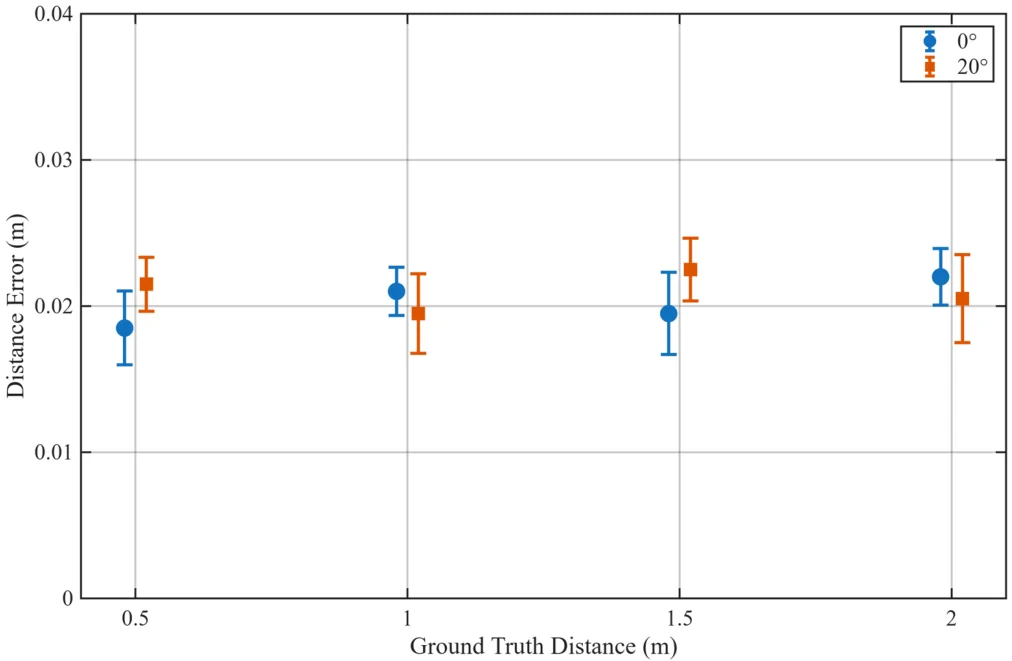
Mean and standard deviation of distance estimation errors according to ground truth distance.

**Figure 9:**
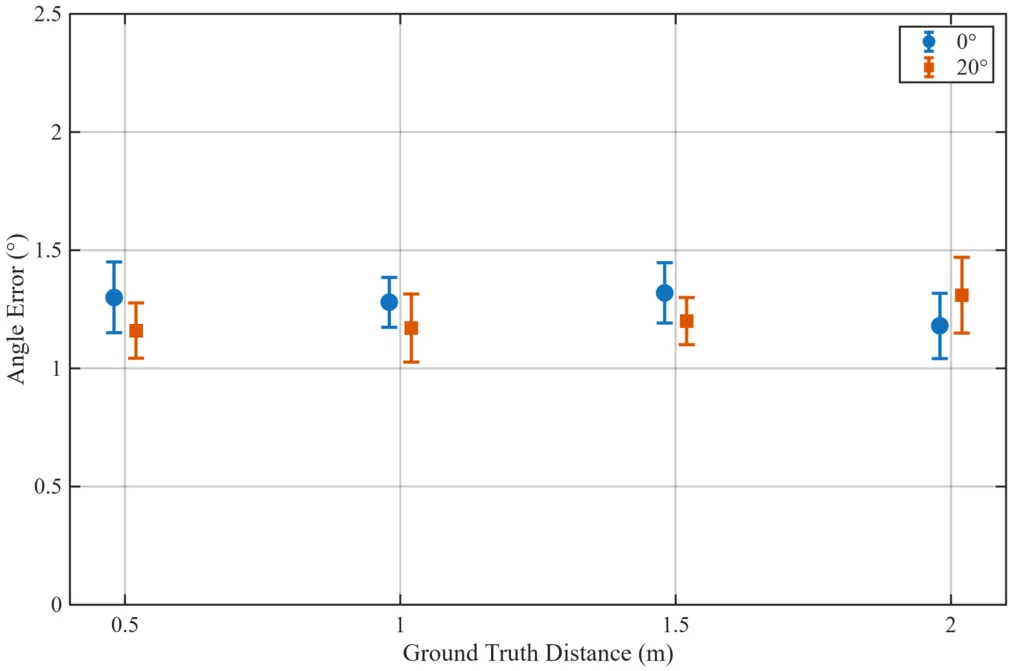
Mean and standard deviation of angle estimation errors according to ground truth distance.

Figure 8 and Figure 9 show the mean and standard deviation of distance error and angle error, respectively, across repeated measurements at each condition. Each point is the mean error from ten repeated measurements, and the error bars show the standard deviation.

In Figure 8, the standard deviation of distance error ranged from 0.002 to 0.003 m at both 0° and 20°, a similar level at both angles, with no consistent increase or decrease in mean error as the set distance increased.

In Figure 9, the standard deviation of angle error ranged from 0.11° to 0.15° at 0° and from 0.10° to 0.16° at 20°. As with distance error, angle error showed no consistent trend with distance, and both angle conditions gave a similar mean error and standard deviation.

Overall, distance and angle error remained within a similar range at both 0° and 20°, with no clear increase as distance grew. These results show that the camera–LiDAR fusion used in this study can repeatedly estimate a person’s position within the distance and angle range tested.

### 3.2. Position Estimation During Navigation

Table 5 reports the difference between the person’s position estimated by camera–LiDAR fusion (the first estimate in each trial) and the tape-measured position used as ground truth in the navigation analysis. The estimate was logged in every trial, including the Conventional trials, in which it was not used by the costmap. The mean distance difference ranged from 0.05 to 0.13 m across the conditions, which is larger than the 0.02 m obtained in the static validation (Table 3 and Table 4). The mean differences ranged from −0.05 to +0.07 m in *x* and from −0.06 to +0.09 m in *y*. Possible contributors are differences in the body surface that the LiDAR returns for different postures, the selected LiDAR return point, and deviations in the robot’s estimated pose, but these factors were not separated in the experiments. This difference is the reason why the navigation results below are evaluated against tape-measured positions and not against the fusion estimate.

### 3.3. Human-Aware Navigation Validation

#### 3.3.1. Single-Target Navigation Results

**Figure 10:**
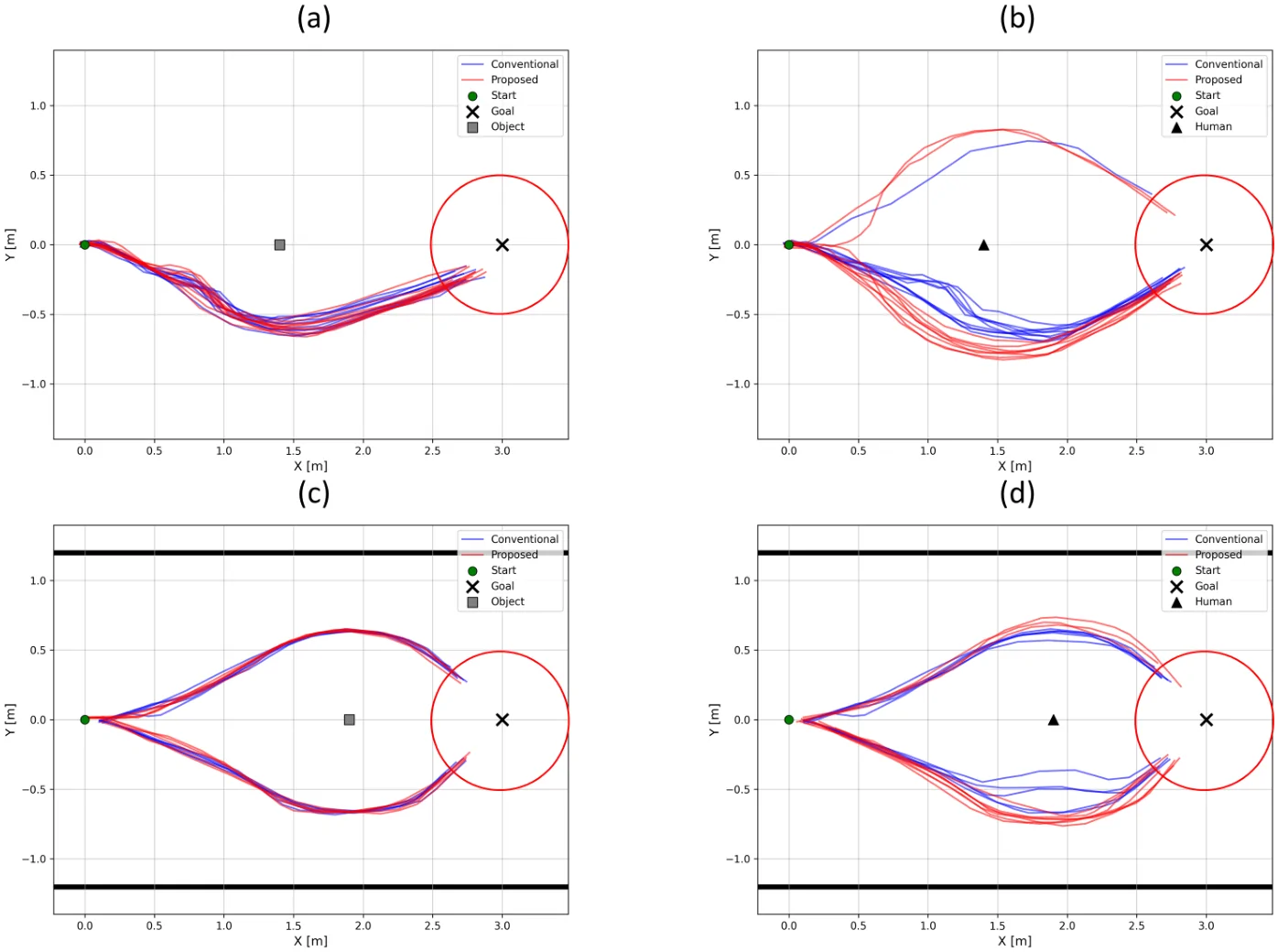
Comparison of robot trajectories between Conventional and Proposed under single-target conditions: (**a**) Object in the open environment, (**b**) Human in the open environment, (**c**) Object in the narrow environment, and (**d**) Human in the narrow environment. The trajectories of the successful trials are overlaid. The red circle around the goal marks the 0.5 m tolerance used to judge navigation complete, and the solid black lines in (**c**,**d**) mark the two side walls of the experimental space.

To compare the driving behavior of Conventional and Proposed under single-target conditions, Figure 10 shows the driving trajectories recorded in the open and narrow environments. Figure 10a,b show the object and human conditions in the open environment, and Figure 10c,d show the same conditions in the narrow environment. The trajectories of the successful trials are overlaid for each condition, and in the narrow-environment plots the solid black lines mark the two side walls of the experimental space. All trials started from the same position, and trajectories were recorded from the robot’s position as estimated by AMCL. Because this position estimate is updated and logged at discrete intervals, a trajectory’s first recorded point may not exactly match the robot’s actual starting position, and its last recorded point may likewise differ slightly from the robot’s position when Nav2 registered the goal as reached.

In Figure 10, the red circle around the goal marks the 0.5 m tolerance used to judge navigation complete. Because of this, the last recorded point of some successful trials falls outside this circle, but Nav2 still reported these trials as succeeded.

For the object condition, shown in Figure 10a,c, Conventional and Proposed produced largely similar trajectories. For the human condition, shown in Figure 10b,d, Proposed kept a greater distance from the person than Conventional. In the open environment, the robot passed the target on one side in nearly every trial, whereas in the narrow environment, shown in Figure 10c,d, it passed on either side across repeated trials. With driving space limited in the narrow environment, the robot passed both sides of the target over repeated trials, likely because small variations in position estimation and in the costmap from one trial to the next shifted which side the planner chose. Videos of the physical trials corresponding to these trajectories are provided as Video S1 in the Supplementary Materials. Table 6 and Table 7 report the success count and the mean and standard deviation of navigation time, path length, and minimum distance to the target in each environment.

In the open environment, the minimum distance to the object was 0.577 m for both Conventional and Proposed. For the person, the minimum distance was 0.499 m under Conventional and 0.739 m under Proposed, an increase of about 0.24 m (48.1%). Path length and navigation time for the person condition also rose under Proposed, to 3.173 m and 20.402 s, compared with 3.028 m and 19.197 s under Conventional.

For the object condition, the *p*-values for navigation time, path length, and minimum distance were 0.766, 0.284, and 1.000, respectively, showing no statistically significant difference between Conventional and Proposed on any metric. For the person condition, the *p*-values for navigation time and path length were 0.002 and 0.003, and the *p*-value for minimum distance was below 0.001, so all three metrics differed significantly between the two methods.

In the narrow environment, the minimum distance to the object was 0.654 m under Conventional and 0.633 m under Proposed (p=0.287). Navigation time rose from 17.388 s under Conventional to 18.697 s under Proposed, and path length rose from 2.740 m to 2.855 m, with *p*-values of 0.010 and 0.038. Since human cost was not applied in the object condition and the minimum distance showed no significant difference, this increase in navigation time and path length cannot be directly attributed to human cost.

For the person condition, the minimum distance was 0.584 m under Conventional and 0.717 m under Proposed, an increase of about 0.13 m (22.8%, p=0.0015). Proposed’s path length and navigation time also rose, to 2.864 m and 18.647 s, compared with 2.707 m and 17.347 s under Conventional (p=0.014 and 0.002).

Taken together, the object condition showed no significant difference in minimum distance between Conventional and Proposed in either environment (p=1.000 and 0.287), while the person condition showed an increase of about 0.24 m in the open environment and 0.13 m in the narrow environment under Proposed (p<0.001 and p=0.0015). Path length increased by about 4.8% and 5.8% and navigation time by about 6.3% and 7.5%. Because navigation time and path length also differed significantly for the object condition in the narrow environment, these two increases cannot be attributed to human cost alone. The significant increase in the minimum distance to a person, together with the absence of a significant change for the object, indicates that the proposed method secures extra distance specifically from people.

To compare the geometric margin between the robot and the target, Table 8 and Table 9 report the worst case, the mean and minimum clearance, and the numbers of overlaps and near-misses. Each count refers to the successful trials of the condition.

In the open environment, the object condition gave similar worst-case distances under the two methods (0.516 m and 0.531 m); under Proposed all ten trials were near-misses for the object, with a minimum clearance of 0.000 m. For the person, the worst-case distance was 0.357 m under Conventional and 0.649 m under Proposed. All nine successful Conventional trials had an overlap, whereas Proposed had no overlap but three near-misses, and its minimum clearance was 0.030 m.

In the narrow environment, the worst-case distance to the object was 0.636 m under Conventional and 0.534 m under Proposed, with two near-misses under Proposed and none under Conventional. For the person, the worst-case distance was 0.385 m under Conventional and 0.664 m under Proposed. Conventional had four overlaps and six near-misses, and Proposed had no overlap but five near-misses, with a minimum clearance of 0.045 m.

These results show that, in both environments, Proposed removed the overlaps that Conventional produced around a person, but its clearance to a person was still below 0.10 m in some trials. Negative clearance was obtained in some trials, but no physical contact between the robot and a target was observed during the trials. This is consistent with the conservative circle approximation, and an overlap should not be read as a collision. The clearance values depend on the position used for the person, and the differences between the fusion estimate and the tape-measured position are given in Table 5.

#### 3.3.2. Multi-Target Navigation Results

**Figure 11:**
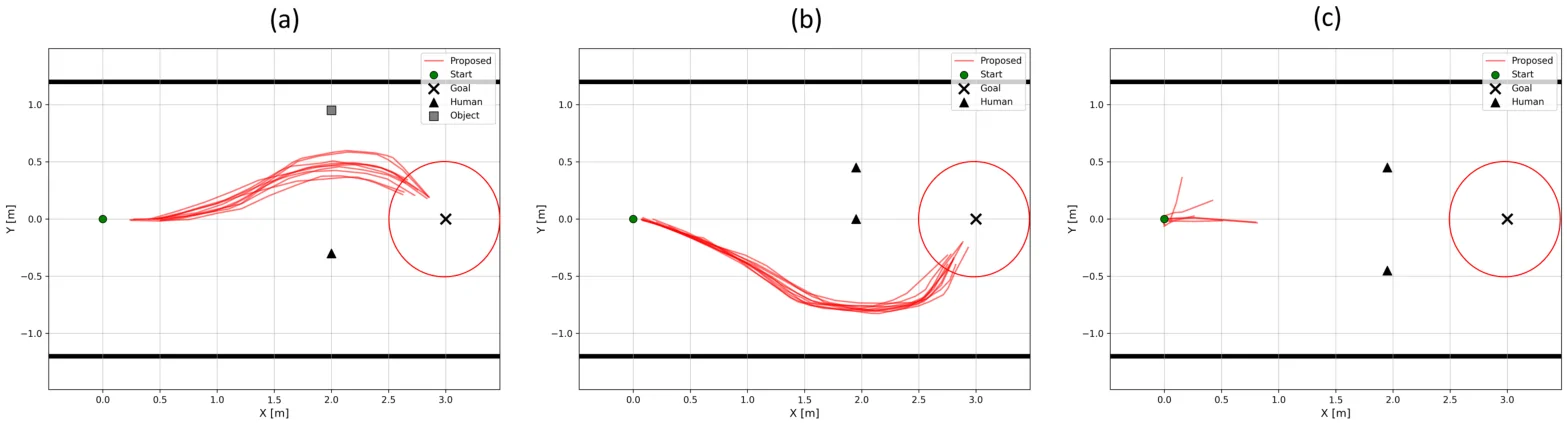
Navigation trajectories in the multi-target experiments: (**a**) Human-Object, (**b**) Human-Human with 0.45 m spacing, and (**c**) Human-Human with 0.90 m spacing. The red circle around the goal marks the 0.5 m tolerance used to judge navigation complete, and the solid black lines mark the two side walls of the experimental space.

To examine how human cost affects the driving path under multi-target conditions, Figure 11 shows the trajectories recorded for the human-object and human-human conditions. Figure 11a shows the human-object condition, and Figure 11b,c show the human-human condition with center-to-center spacing of 0.45 m and 0.90 m. Trajectories were recorded from AMCL-based position estimates as in Section 3.3.1, and the red circle around the goal in Figure 11 marks the same 0.5 m navigation-complete tolerance. The markers show the tape-measured positions of the targets. As before, a trajectory’s first and last recorded points may differ slightly from the robot’s actual start position and from its position when Nav2 registered the goal as reached.

In the human-object condition shown in Figure 11a, the robot passed through the space between the person and the object, with its path closer to the object than to the midpoint between the two targets. In the human-human condition with 0.45 m spacing, shown in Figure 11b, the robot avoided passing between the two people and went around them on the side with more free width (y<0), completing navigation to the goal. In the human-human condition with 0.90 m spacing, however, navigation was not completed, and the trials failed in different ways: the robot stopped mid-course or rotated in place in some trials, and failed to move from the starting position in others. Figure 11c therefore shows only the movement recorded before each trial was terminated. Table 10 reports the quantitative driving results for each multi-target condition.

In the human-object condition, the minimum distance was 0.761 m from the person and 0.485 m from the object, about 0.28 m greater for the person. Under the same driving conditions, the robot therefore kept a greater distance from the person, who had human cost applied, than from the ordinary object. In the human-human condition with 0.45 m spacing, the minimum distance from either person was 0.774 m, and navigation to the goal was completed, with a navigation time of 20.750 s and a path length of 3.079 m. This shows that navigation remains possible when human cost is applied to two people.

In the human-human condition with 0.90 m spacing, navigation was not completed in any trial, so no representative values are reported. The difference between the two spacings can be examined from the geometry of the passage, which is 2.40 m wide, with the walls at y=±1.2 m. The robot is 0.42 m wide, and each person is modeled as a circle of radius 0.30 m. At 0.90 m spacing, the gap between the two model circles is 0.90−2×0.30=0.30 m, which is narrower than the robot, so passing between the two people is infeasible in the model. On the outside, the gap between a model circle and the wall is 1.2−(0.45+0.30)=0.45 m on both sides, only 0.03 m wider than the robot. With 0.45 m spacing, the people stood at y=0.45 m and y=0.00 m. The gap between the model circles is negative, so the pair acts as one obstacle. On the side of y>0 the gap to the wall is again 0.45 m, whereas on the side of y<0 it is 1.2−0.30=0.90 m, more than twice the robot width. The robot used this wider side (Figure 11b). The 0.03 m margin at 0.90 m spacing is smaller than the position differences in Table 5, and the inflated cost around the people and the walls overlaps in these gaps, so a feasible path is practically unavailable to the planner. Measured on the bodies, which are about 0.20 m in half-width, the gap between two people at 0.90 m spacing is about 0.50 m, wider than the robot, so the failure comes from the conservative model and the cost settings and not from a physically closed passage.

Table 11 shows that, in the human-object condition, the robot gave up clearance to the object in favor of the person. The clearance to the person was positive in all trials, whereas the clearance to the object was reduced. No contact was observed by the experimenters. Keeping a larger distance from a person can bring the robot closer to a nearby ordinary obstacle. In the human-human condition with 0.45 m spacing, there was no overlap and no near-miss, with a minimum clearance of 0.115 m.

### 3.4. Comparison with Uniform Inflation

Table 12 compares Proposed with Conventional using the default inflation radius (0.45 m) and with Uniform Inflation (0.78 m) in the four single-target conditions.

In the open environment with a person, Uniform Inflation reached a minimum distance of 0.710±0.125 m, which did not differ significantly from that of Proposed (0.739±0.053 m, p=0.58). It was achieved with a lower success rate (7/10 against 9/10 for Proposed) and with a longer path and time (3.596 m and 26.086 s, against 3.173 m and 20.402 s). The same inflation also increased the distance to the object from 0.577 m to 0.801 m, so the added clearance was not specific to people. In the narrow environment, Uniform Inflation succeeded in only 4 of 10 trials with a person, and the minimum distance to the person (0.534 m) was lower than under Proposed (0.717 m, p=0.018). The distance to the object also decreased relative to the default radius (0.608 m against 0.654 m, with 8/10 successes). A plausible reason is that the enlarged inflated regions of the two walls and the target overlap in the 2.4 m passage and leave little cost-free space. The causes of the failed trials were not analyzed. The inflation radius was tuned only to the open-environment person condition, and other values were not explored, so this comparison shows that uniform inflation was not an equivalent substitute at the tested setting. The comparison supports selective clearance relative to this one uniformly enlarged setting. It does not establish an advantage over human-specific inflation or semantic costmap approaches, which were not implemented here (Section 4.2).

### 3.5. Inflation Sweep in the Two-Person Passage

Table 13 shows the effect of reducing the inflation radius in the human-human condition with 0.90 m spacing, in which Proposed failed in all ten trials with the default 0.45 m radius.

The robot completed 1 of 5 trials with an inflation radius of 0.40 m, 2 of 5 with 0.35 m, and all five with 0.30 m. The failed trials ended with the Nav2 result ABORTED. With 0.40 m, three trials stopped in front of the people (one after a short advance, one at about 1.2 m, and one without moving), and one trial passed between the people but stopped before reaching the goal. With 0.35 m, all three failed trials stopped in front of the people, one of them without moving. These results show that the failure at 0.90 m spacing depends on the inflation setting and not on the perception. Passability was gained by reducing the inflation radius, which also reduces the margin kept to the people. The 0.30 m body model is a modeling radius (Section 2.2.4) and is larger than the half-width of the body observed by the LiDAR (about 0.20 m), so it is a conservative approximation. With this model, the gap between two people at 0.90 m spacing is 0.30 m, whereas it would be about 0.50 m with a radius of 0.20 m, and the clearances in Table 8, Table 9, Table 10 and Table 11 are 0.10 m smaller per person than with a radius of 0.20 m. The larger radius thus errs on the side of clearance and, at the default inflation radius, makes the 0.90 m passage impassable for the planner. No physical contact occurred in these trials.

**Figure 12:**
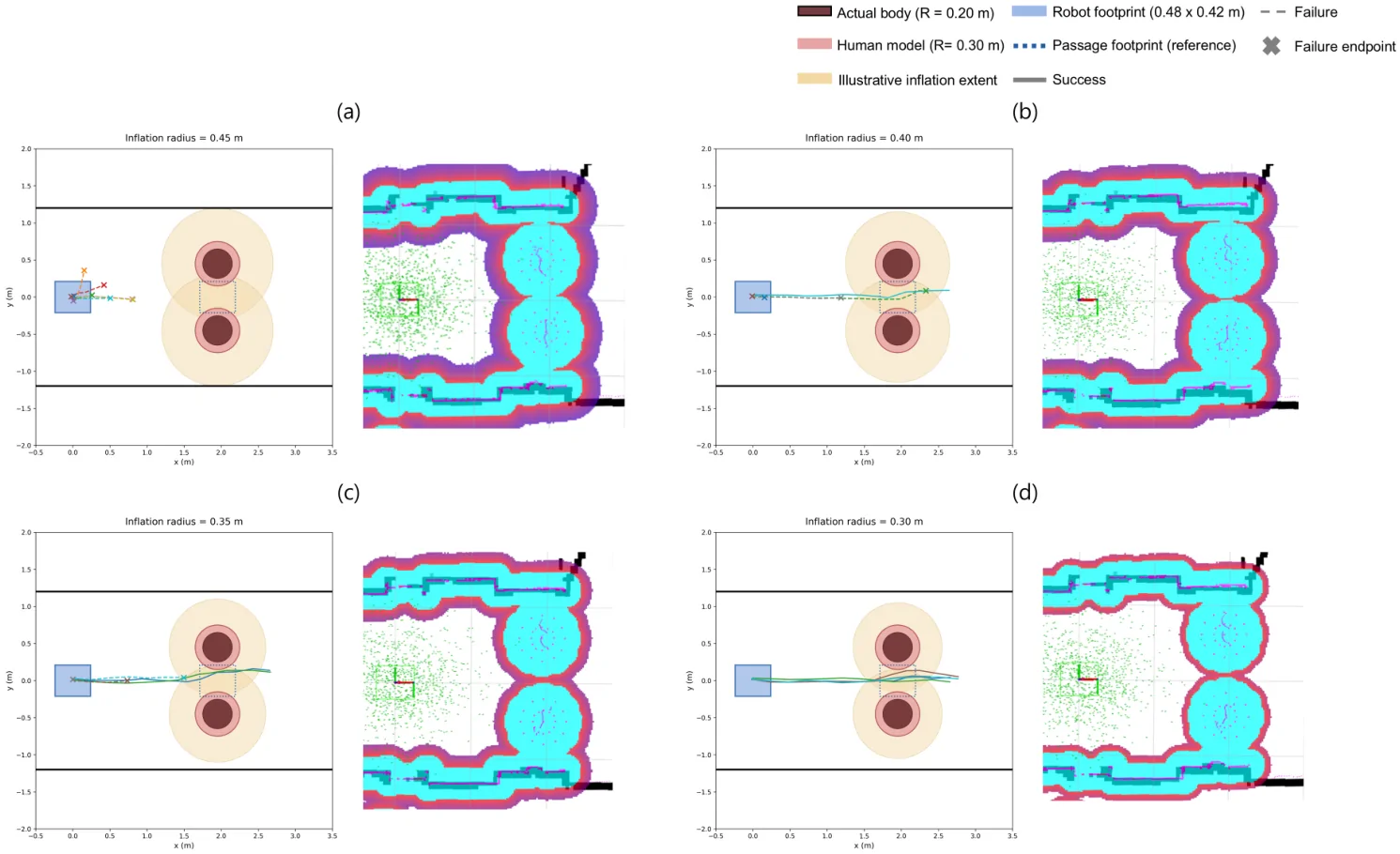
Two-person passage (0.90 m spacing, passage width 2.4 m) with inflation radii of (**a**) 0.45 m, (**b**) 0.40 m, (**c**) 0.35 m, and (**d**) 0.30 m. In each panel, the left plot is a drawing to scale in the map frame. The thick black lines are the side walls (y=±1.2 m). The dark red circles are the actual bodies (R=0.20 m), the light red circles are the body models (R=0.30 m), and the beige circles show the illustrative extent of the inflation. The blue rectangle is the robot footprint (0.48×0.42 m) at the start position, and the dotted rectangle is the same footprint placed midway between the people for reference. Each colored line is the recorded trajectory of one trial: solid lines are successful trials, dashed lines are failed trials, and × marks the end point of a failed trial. The right image is the local costmap captured in RViz with the same inflation radius.

The two statements about the 0.90 m case refer to different quantities and are reconciled as follows. Figure 12 shows the geometry. With the default inflation radius (0.45 m), the two body models (R=0.30 m, centers 0.90 m apart) leave a gap of 0.30 m, which is narrower than the 0.42 m width of the robot, and the cost regions of the two people overlap in the gap. The successful passages in Table 13 were obtained with the same body models. They therefore occurred where the model says that the robot does not fit: the circumscribed circle of the robot overlapped the body models, which is a negative model clearance, and no physical contact was observed. The body model is conservative. Two bodies with a half-width of about 0.20 m leave a physical gap of about 0.50 m, which is wider than the robot. What changed between the failed and the successful trials is the inflation cost around the body models. The local costmaps captured in RViz (Figure 12) show that the inflated region around the two people becomes smaller as the radius is reduced. We did not analyze cell by cell which costs blocked the planner.

### 3.6. Computational Load

Table 14 lists the CPU and memory use of each component, and Table 15 lists the publication rates of the main topics, measured during navigation with Proposed. The whole system used 83.6% of the CPU on average, with a maximum of 99%, and about 3 GiB of the 16 GB memory of the computer. The YOLOv8n and SORT process dominated with a mean of 233.6%, where the percentage is summed over the cores of the four-core CPU (about 58% of its total capacity). The system-wide value also includes processes that are not listed in the table.

Detection and tracking ran at about 7.6 Hz, roughly half of the camera frame rate, so the person’s position was updated about every 0.13 s. The person points were published faster than the tracking output, and the costmaps were published at lower rates, which are publication rates and not internal update rates. These rates do not give the end-to-end latency, which was not measured. The high CPU load leaves little margin on this computer for additional processing.

## 4. Discussion

### 4.1. Interpretation of the Results

The results show that representing a person as a body-sized obstacle in the standard Obstacle Layer, and removing the person’s own LiDAR returns, increases the distance kept from the person without changing the distance kept from ordinary objects. The effect does not come from a person-specific inflation parameter. The Inflation Layer uses the same radius and scaling factor for all obstacles, and it acts on a larger footprint when the obstacle is a person. The extent of about 0.75 m from the person’s center is comparable to the personal distances described in the proxemics literature [24] and to the passing distances studied for social robots [25], but it was not tuned to a social criterion in this study.

Uniform inflation did not provide the same effect. Enlarging the inflation radius for all obstacles gave a similar person distance in the open environment, but it also moved the robot away from objects, lowered the success rate, and lengthened the path, and in the narrow passage the distance to the person (0.534 m) remained below that of Proposed (0.717 m). This supports the use of a person-specific footprint, but it is a comparison with one baseline setting, and it does not establish an advantage over human-specific inflation, semantic costmap, or dedicated social-navigation approaches, which were not evaluated.

The added clearance has costs. Path length and navigation time increased by about 5% and 7% for a person, although similar increases also occurred for the object in the narrow environment. The robot also accepted a smaller margin to an ordinary obstacle that was near a person (human-object condition). In confined spaces, the conservative body model can make a passage unusable, as in the 0.90 m case, where passage became possible only after the inflation radius was reduced. The choice of the body-model radius (R=0.30 m), which exceeds the measured half-width of the body (about 0.20 m), and of the inflation radius is therefore a trade-off between clearance and passability. A smaller body-model radius was not tested.

### 4.2. Comparison with Related Studies

Table 16 summarizes studies that bring a person into a costmap or a navigation planner. Three groups can be distinguished. The first adds a dedicated social-cost layer to the layered costmap [5], for example Gaussian proxemic zones in a Nav2 layer [7], age- and group-based layers [23], and speed- or head-pose-dependent social-distance regions [9,12]. The second predicts pedestrian trajectories and builds time-dependent cost or occupancy maps [8,19,26]. The third acts at the level of decision making [7,20]. The proposed method belongs to the first group in purpose, but it uses no dedicated layer.

A direct numerical comparison with these studies is not possible, for five reasons. (i) The sensing differs: RGB-D cameras, LiDAR, camera–LiDAR fusion, and positions supplied by a simulator. (ii) The scenarios differ: simulated or real, stationary or moving people, children, and groups, whereas the present study used stationary adults. (iii) The metric definitions differ: minimum distance, invasion distance, collision rate, and social-compliance indicators are used, and the person position used as the reference (estimated or measured) is not always stated. (iv) The baselines differ: a traditional costmap, learning-based planners, or none. (v) Computation is rarely reported, and the hardware differs.

The proposed method differs from these approaches in what it changes. It adds no layer, plugin, or planner. It uses the standard Obstacle Layer and Inflation Layer with unchanged parameters, and only the footprint of the person and the removal of the person’s own scan returns differ from conventional Nav2. In terms of purpose, it is still a human-specific cost; the difference lies in how it is implemented. Its cost field is also less expressive. It does not use speed, orientation, age, or group membership, so the approaches in Table 16 are expected to suit moving or interacting people better. Our experiments do not show that the proposed method outperforms any of these approaches or a human-specific inflation radius. Its demonstrated value is limited to a simple way of giving a stationary person a larger clearance in a standard Nav2 configuration, with selectivity relative to one uniform-inflation setting.

### 4.3. Limitations

This study has the following limitations. (i) All people and objects were stationary, so the method was not evaluated with moving people, and the person’s motion is not used in planning. (ii) One robot platform, one indoor passage, and ten trials per condition (five in the inflation sweep) were used, and comparison with dedicated social-navigation planners was not performed. (iii) The conditions were tested in a fixed order, with Conventional trials first and Proposed trials second in the same session, without randomization. (iv) No extrinsic calibration between the camera and the LiDAR was performed. The position differences during navigation (0.05–0.13 m) were larger than in the static validation (about 0.02 m), and the clearance values are therefore best read as relative comparisons. (v) Overlap and clearance are computed from circumscribed circles. They are conservative, and they are not contact measurements; contact was checked visually and not recorded on video in the main experiments. (vi) Four of the 80 single-target trials of Conventional and Proposed ended with an ABORTED result, and their causes were not identified. (vii) The end-to-end latency was not measured, and the CPU load was high on the CPU-only on-board computer. (viii) Detection dropouts make the person fall back to an ordinary obstacle for the affected frames, and this behavior was not evaluated separately. (ix) The uniform-inflation baseline used one radius, which was matched to Proposed in the open person condition and not tuned elsewhere. (x) No human-specific inflation, semantic costmap, or social-navigation planner was implemented, so the comparison with these approaches is limited to the literature review in Section 4.2. (xi) The intended applications are not demonstrated, because the validation used stationary people in a laboratory. (xii) The body radius is a fixed modeling value of 0.30 m, and it does not adapt to the body width of the person observed by the LiDAR.

## 5. Conclusions

This study proposes a human-aware navigation method that uses a low-cost monocular RGB camera and 2D LiDAR to make a standard ROS2 Nav2 configuration treat a person differently from an ordinary obstacle. A person is detected and tracked with YOLOv8n and SORT, localized through camera–LiDAR fusion, represented as a body-sized set of virtual obstacle points in the Obstacle Layer, and removed from the LiDAR scan, while the Inflation Layer and the rest of the navigation structure are unchanged.

In single-target experiments with tape-measured ground truth, the minimum distance to a person increased from 0.499 m to 0.739 m in the open environment and from 0.584 m to 0.717 m in the narrow environment, compared with conventional Nav2, whereas the minimum distance to an object did not change significantly. Overlaps of the circumscribed circles that occurred around a person under Conventional were removed under Proposed, although the clearance to a person was still below 0.10 m in some trials. Path length increased by about 4.8–5.8% and navigation time by about 6.3–7.5% for a person. At the one setting tested, enlarging the inflation radius uniformly to 0.78 m gave a similar person distance in the open environment, but it also increased the distance to objects, reduced the success rate, lengthened the path, and did not increase the person distance in the narrow passage. In multi-target experiments, the robot kept a greater distance from the person than from an object, but it did so at the expense of a small margin to the object. With two people 0.90 m apart in a 2.4 m passage, the robot could not pass, and reducing the inflation radius enabled passage only with negative clearance.

These findings are limited to stationary people in a laboratory, one platform, and one passage. They do not show an advantage over existing human-specific costmap approaches. Future work should extend the method to moving pedestrians, evaluate it against dedicated social-navigation planners, adapt the body model and the inflation to the available space, estimate the body width of each person from the LiDAR scan and adjust the human cost accordingly, and measure the end-to-end latency.

## Figures and Tables

**Table 1:** Hardware components and specifications.

Component	Model	Details
On-board Computer	ATEC Desktop PC	CPU: Intel i5-6500; GPU: Intel UHD Graphics 530; RAM: DDR4 16 GB; NIC: Intel I210; OS: Ubuntu 20.04
2D LiDAR	RPLIDAR A1	Detection angle: 360°; Detection distance: 12.0 m; Scanning frequency: 10.0 Hz
Monocular RGB Camera	Logitech StreamCam	Resolution: 320 × 240; Field of view (FOV): 78° (diagonal)
Motor	EzM2-86M-A	Encoder resolution: 10,000 pulse/rev
Motor Driver	EzS2-EC-86M-A	Closed-loop step motor driver

**Table 2:** Experimental conditions for single-target navigation validation.

Scenario	Environment	Target	Method
Scenario 1	Open environment	Object Human	Conventional/Proposed
Scenario 2	Narrow environment	Object Human	Conventional/Proposed

**Table 3:** Mean and standard deviation of distance and angle errors at 0°.

Ground Truth Distance (m)	Mean Estimated Distance (m)	Mean Estimated Angle (°)	Distance Error (m) (Mean ± SD)	Angle Error (°) (Mean ± SD)
0.5	0.494	0.24	0.019 ± 0.003	1.30 ± 0.15
1.0	1.008	−0.18	0.021 ± 0.002	1.28 ± 0.11
1.5	1.495	0.31	0.020 ± 0.003	1.32 ± 0.13
2.0	2.008	−0.27	0.022 ± 0.002	1.18 ± 0.14

**Table 4:** Mean and standard deviation of distance and angle errors at 20°.

Ground Truth Distance (m)	Mean Estimated Distance (m)	Mean Estimated Angle (°)	Distance Error (m) (Mean ± SD)	Angle Error (°) (Mean ± SD)
0.5	0.507	19.78	0.022 ± 0.002	1.16 ± 0.12
1.0	0.995	20.26	0.020 ± 0.003	1.17 ± 0.14
1.5	1.508	19.81	0.023 ± 0.002	1.20 ± 0.10
2.0	1.993	20.33	0.021 ± 0.003	1.31 ± 0.16

**Table 5:** Difference between the fusion-estimated and tape-measured person positions during navigation (estimate minus ground truth, mean ± SD over all ten trials per row, including trials that did not succeed).

Condition	Method	Ground Truth (x,y) (m)	Δ*x* (m)	Δ*y* (m)	Distance (m)
Open, human	Conventional	(1.40, 0.00)	+0.021 ± 0.022	+0.088 ± 0.058	0.095 ± 0.054
Open, human	Proposed	(1.40, 0.00)	+0.011 ± 0.018	+0.048 ± 0.025	0.052 ± 0.026
Narrow, human	Conventional	(1.90, 0.00)	+0.030 ± 0.087	−0.010 ± 0.109	0.131 ± 0.037
Narrow, human	Proposed	(1.90, 0.00)	−0.016 ± 0.054	+0.040 ± 0.080	0.088 ± 0.052
Human-Object, human	Proposed	(2.00, −0.30)	−0.051 ± 0.063	+0.048 ± 0.075	0.106 ± 0.048
Human-Human 0.45 m, person 1	Proposed	(1.95, 0.45)	−0.001 ± 0.032	−0.046 ± 0.034	0.057 ± 0.030
Human-Human 0.45 m, person 2	Proposed	(1.95, 0.00)	+0.060 ± 0.062	−0.057 ± 0.042	0.102 ± 0.041
Human-Human 0.90 m, person 1	Proposed	(1.95, 0.45)	0.000 ± 0.027	−0.047 ± 0.023	0.054 ± 0.024
Human-Human 0.90 m, person 2	Proposed	(1.95, −0.45)	+0.066 ± 0.048	−0.052 ± 0.031	0.099 ± 0.020

**Table 6:** Navigation results of the single-target experiments in the Open Environment (mean ± SD over successful trials; minimum distance to the tape-measured target position).

Target	Method	Success	Navigation Time (s)	Path Length (m)	Minimum Distance (m)
Object	Conventional	9/10	19.325 ± 0.368	2.988 ± 0.082	0.577 ± 0.042
Object	Proposed	10/10	19.387 ± 0.520	3.029 ± 0.079	0.577 ± 0.028
Human	Conventional	9/10	19.197 ± 0.552	3.028 ± 0.091	0.499 ± 0.099
Human	Proposed	9/10	20.402 ± 0.813	3.173 ± 0.081	0.739 ± 0.053

**Table 7:** Navigation results of the single-target experiments in the Narrow Environment (mean ± SD over successful trials; minimum distance to the tape-measured target position).

Target	Method	Success	Navigation Time (s)	Path Length (m)	Minimum Distance (m)
Object	Conventional	10/10	17.388 ± 0.843	2.740 ± 0.125	0.654 ± 0.014
Object	Proposed	9/10	18.697 ± 1.080	2.855 ± 0.097	0.633 ± 0.054
Human	Conventional	10/10	17.347 ± 0.831	2.707 ± 0.132	0.584 ± 0.095
Human	Proposed	10/10	18.647 ± 0.802	2.864 ± 0.125	0.717 ± 0.031

**Table 8:** Clearance analysis in the Open Environment (tape-measured target positions). Overlap and near-miss are model-based; no physical contact was observed.

Condition	Success	Worst Case (m)	Mean Clearance (m)	Min. Clearance (m)	Model Overlap	Model Near-Miss
Object, Conventional	9/10	0.516	0.046	−0.015	1	7
Object, Proposed	10/10	0.531	0.046	0.000	0	10
Human, Conventional	9/10	0.357	−0.120	−0.262	9	0
Human, Proposed	9/10	0.649	0.120	0.030	0	3

**Table 9:** Clearance analysis in the Narrow Environment (tape-measured target positions). Overlap and near-miss are model-based; no physical contact was observed.

Condition	Success	Worst Case (m)	Mean Clearance (m)	Min. Clearance (m)	Model Overlap	Model Near-Miss
Object, Conventional	10/10	0.636	0.123	0.105	0	0
Object, Proposed	9/10	0.534	0.102	0.003	0	2
Human, Conventional	10/10	0.385	−0.035	−0.234	4	6
Human, Proposed	10/10	0.664	0.098	0.045	0	5

**Table 10:** Navigation results in the multi-target experiments (mean ± SD over successful trials; distances to the tape-measured target positions).

Target	Navigation Time (s)	Path Length (m)	Min. Human Distance (m)	Min. Object Distance (m)	Success
Human-Object	16.471 ± 1.297	2.494 ± 0.232	0.761 ± 0.058	0.485 ± 0.073	10/10
Human-Human (0.45 m)	20.750 ± 1.521	3.079 ± 0.135	0.774 ± 0.022	-	10/10
Human-Human (0.90 m)	-	-	-	-	0/10

**Table 11:** Clearance analysis in the multi-target experiments (tape-measured target positions). Overlap and near-miss are model-based; no physical contact was observed.

Condition	Success	Worst Case (m)	Mean Clearance (m)	Min. Clearance (m)	Model Overlap	Model Near-Miss
Human-Object (Object)	10/10	0.367	−0.046	−0.164	8	2
Human-Object (Human)	10/10	0.674	0.142	0.055	0	2
Human-Human (0.45 m)	10/10	0.734	0.155	0.115	0	0
Human-Human (0.90 m)	0/10	-	-	-	-	-

**Table 12:** Comparison of Conventional (inflation radius 0.45 m), Uniform Inflation (0.78 m), and Proposed in the single-target conditions (mean ± SD over successful trials).

Condition	Method	Success	Time (s)	Path (m)	Min. Distance (m)	Min. Clearance (m)
Open, object	Conventional	9/10	19.325 ± 0.368	2.988 ± 0.082	0.577 ± 0.042	−0.015
	Uniform Inflation	8/10	24.765 ± 1.026	3.668 ± 0.116	0.801 ± 0.119	0.131
	Proposed	10/10	19.387 ± 0.520	3.029 ± 0.079	0.577 ± 0.028	0.000
Open, human	Conventional	9/10	19.197 ± 0.552	3.028 ± 0.091	0.499 ± 0.099	−0.262
	Uniform Inflation	7/10	26.086 ± 3.641	3.596 ± 0.238	0.710 ± 0.125	−0.172
	Proposed	9/10	20.402 ± 0.813	3.173 ± 0.081	0.739 ± 0.053	0.030
Narrow, object	Conventional	10/10	17.388 ± 0.843	2.740 ± 0.125	0.654 ± 0.014	0.105
	Uniform Inflation	8/10	24.487 ± 2.644	3.380 ± 0.153	0.608 ± 0.085	−0.091
	Proposed	9/10	18.697 ± 1.080	2.855 ± 0.097	0.633 ± 0.054	0.003
Narrow, human	Conventional	10/10	17.347 ± 0.831	2.707 ± 0.132	0.584 ± 0.095	−0.234
	Uniform Inflation	4/10	24.357 ± 1.215	3.321 ± 0.089	0.534 ± 0.082	−0.185
	Proposed	10/10	18.647 ± 0.802	2.864 ± 0.125	0.717 ± 0.031	0.045

**Table 13:** Effect of the inflation radius in the human-human condition with 0.90 m spacing (Proposed).

Inflation Radius (m)	Success
0.45 (default)	0/10
0.40	1/5
0.35	2/5
0.30	5/5

**Table 14:** CPU and memory use of the software components during navigation with Proposed (120 s mean; CPU percentages are summed over cores, so one core corresponds to 100%).

Component	Mean CPU (%)	Memory (MiB)
Camera	0.43	86.3
YOLOv8n + SORT	233.55	640.8
Camera–LiDAR fusion	0.73	29.8
RPLIDAR	1.08	27.7
AMCL	0.32	36.7
Nav2 controller	2.12	61.3
Nav2 planner	0.61	45.2

**Table 15:** Publication rates of the main topics along the processing chain.

Stage	Topic	Rate (Hz)
Camera input	/image_raw	14.97
YOLOv8n + SORT output	/tracked_bboxes	7.63
Person position	/locked_person_center	13.64
Virtual obstacle points	/person_points	13.72
Local costmap	/local_costmap/costmap	3.13
Global costmap	/global_costmap/costmap	0.64
Velocity command	/cmd_vel	10.18

**Table 16:** Summary of related studies. Entries are taken from the cited papers. n.r.: not reported in the text reviewed. For [8,20], only the abstract was reviewed.

Study	Sensing	Human Cost/Navigation Method	Scenarios	Computation	Reported Performance
Ginés Clavero et al. [7]	Person pose from the simulator; OpenPose- based detection on the real robot (input sensor n.r.)	Nav2 social layer with asymmetric Gaussian proxemic zones; zones adapt to activity, velocity, and requests; behavior tree or PDDL decision making; TEB or DWB	Simulated person, 100 iterations per controller; one real-robot repeat in a smaller space	CPU about 7–14% and RAM 3.0–4.6 GB on the simulation computer; real robot n.r.	Navigation time (about 35 s and 41 s with TEB), time in the personal zone; no comparison with a baseline clearance
Fang et al. [8]	n.r.	Multi-layer dynamic costmaps from predicted pedestrian trajectories, with social cost over time steps; global search planner	Pedestrian and group motion; simulation and a real scene	n.r.	Shorter path and time than a traditional planner and a larger comfort distance, stated qualitatively
Kang et al. [23]	RGB-D (90°) and 2D LiDAR (270°); YOLOv7, SORT	Social type-aware costmap (age-based and group-based layers) as a ROS plugin; Navfn and DWA	Gazebo (5 runs per scenario); 8 real scenarios with adults, children, and groups, stationary and moving	n.r.	Minimum distance to an adult in simulation 0.76 to 1.11 m, with similar time (22.39 to 22.69 s); real stationary adult 0.66 to 1.09 m, against a traditional costmap
Nguyen et al. [12]	2D LiDAR and RGB-D camera on a Jetson Orin NX; Kalman- filter fusion	Social costmap layer with a symmetric (stationary) or asymmetric (moving) Gaussian; DWA	One person, stationary and moving; number of trials n.r.	Hardware named; runtime n.r.	Fusion error about five times smaller than the camera alone; planning results qualitative; no baseline
Kang et al. [9]	2D LiDAR and depth camera; head pose	Social-distance costmap scaled with speed, and a head-pose-based trajectory costmap, in the standard ROS navigation framework	Simulation with varied speed and trajectories; a real-world experiment (details n.r.)	n.r.	Existing methods violated the social distance and the proposed one did not; numbers n.r.
Zhang et al. [19]	n.r.	Dynamic occupancy map from predicted human motion; zero-shot	Social-HM3D and Social-MP3D datasets	n.r. for the method	Human collision rate reduced by over 10% against learning-based planners
Zhang et al. [20]	n.r.	Language-model decision maker for the local controller	Three simulated environments with passing and conversing people	n.r.	Eight metrics; better in most social situations; numbers n.r.
Proposed	Monocular RGB camera and 2D LiDAR; YOLOv8n, SORT	Body-sized virtual obstacle points in the standard Obstacle Layer; person’s scan returns removed; Inflation Layer unchanged	Real robot; stationary people; open and narrow passage; 10 trials per condition	Core i5-6500 (4 cores): CPU 83.6% mean, about 3 GiB RAM; rates in Table 15; latency not measured	Person distance +48.1% (open) and +22.8% (narrow); success rate, clearance, and time reported

## Data Availability

The raw data supporting the conclusions of this article will be made available by the authors on request.

## Supplementary Materials

The following supporting information can be downloaded at: https://www.mdpi.com/article/10.3390/s26217046/s1, Video S1: Videos of the navigation trials of Conventional and Proposed in the single-target and multi-target conditions.
